# Determinants and Outcomes of Suicidal Behavior Among Patients With Major Depressive Disorder

**DOI:** 10.1001/jamapsychiatry.2023.2833

**Published:** 2023-08-16

**Authors:** Johan Lundberg, Thomas Cars, Erik Lampa, Katarina Ekholm Selling, Amy Leval, Anna Gannedahl, Mikael Själin, Carl Björkholm, Clara Hellner

**Affiliations:** 1 Centre for Psychiatry Research, Department of Clinical Neuroscience, Karolinska Institutet, and Stockholm Health Care Services, Stockholm, Sweden; 2 Sence Research AB, Uppsala, Sweden; 3 Department of Medical Sciences, Uppsala University, Uppsala, Sweden; 4 Department of Women’s and Children’s Health, Uppsala University, Uppsala, Sweden; 5 Department of Medical Epidemiology and Biostatistics, Karolinska Institutet, Stockholm, Sweden; 6 Janssen-Cilag AB, Solna, Sweden

## Abstract

**Importance:**

Major depressive disorder (MDD) is an important risk factor of suicidal behavior, but the added burden of suicidal behavior and MDD on the patient and societal level, including all-cause mortality, is not well studied. Also, the contribution of various prognostic factors for suicidal behavior has not been quantified in larger samples.

**Objective:**

To describe the clinical and societal outcomes, including all-cause mortality, of suicidal behavior in patients with MDD and to explore associated risk factors and clinical management to inform future research and guidelines.

**Design, Setting, and Participants:**

This population-based cohort study used health care data from the Stockholm MDD Cohort. Patients aged 18 years or older with episodes of MDD diagnosed between January 1, 2012, and December 31, 2017, in any health care setting were included. The dates of the data analysis were February 1 to November 1, 2022.

**Exposures:**

Patients with MDD with and without records of suicidal behavior.

**Main Outcomes and Measures:**

The main outcome was all-cause mortality. Secondary outcomes were comorbid conditions, medications, health care resource utilization (HCRU), and work loss. Using Region Stockholm registry variables, a risk score for factors associated with suicidal behavior within 1 year after the start of an MDD episode was calculated.

**Results:**

A total of 158 169 unipolar MDD episodes were identified in 145 577 patients; 2240 (1.4%) of these episodes, in 2219 patients, included records of suicidal behavior (mean [SD] patient age, 40.9 [18.6] years; 1415 episodes [63.2%] in women and 825 [36.8%] in men). A total of 11 109 MDD episodes in 9574 matched patients with MDD without records of suicidal behavior were included as controls (mean [SD] patient age, 40.8 [18.5] years; 7046 episodes [63.4%] in women and 4063 [36.6%] in men). The all-cause mortality rate was 2.5 per 100 person-years at risk for the MDD-SB group and 1.0 per 100 person-years at risk for the MDD-non-SB group, based on 466 deaths. Suicidal behavior was associated with higher all-cause mortality (hazard ratio, 2.62 [95% CI, 2.15-3.20]), as well as with HCRU and work loss, compared with the matched controls. Patients with MDD and suicidal behavior were younger and more prone to have psychiatric comorbid conditions, such as personality disorders, substance use, and anxiety, at the start of their episode. The most important factors associated with suicidal behavior within 1 year after the start of an MDD episode were history of suicidal behavior and age, history of substance use and sleep disorders, and care setting in which MDD was diagnosed.

**Conclusions and Relevance:**

This cohort study’s findings suggest that high mortality, morbidity, HCRU, and work loss associated with MDD may be substantially accentuated in patients with MDD and suicidal behavior. Use of medication aimed at decreasing the risk of all-cause mortality during MDD episodes should be systematically evaluated to improve long-term outcomes.

## Key Points

**Question:**

What are the clinical and societal outcomes, including all-cause mortality, associated with suicidal behavior in patients with major depressive disorder (MDD)?

**Findings:**

In this cohort study of 158 169 unipolar MDD episodes, 1.4% involved records of suicidal behavior. The all-cause mortality among patients with suicidal behavior was 2.6 times higher than among matched patients with MDD without records of suicidal behavior.

**Meaning:**

These findings show an association between suicidal behavior and all-cause mortality in patients with MDD and warrant additional interventional studies in health care practice.

## Teaser

This cohort study examines the outcomes and associated risk factors of suicidal behavior in patients with major depressive disorder.

## Introduction

According to the World Health Organization, approximately 5% of the adult population worldwide experienced depression in 2021.^1^ Depression is associated with increased all-cause mortality.^2^ A recent populationwide study showed that all-cause mortality was more than doubled in patients with major depressive disorder (MDD) compared with population controls and that mortality is further increased in patients with treatment-resistant MDD.^3,4^ Suicidal thoughts or behaviors are recognized as diagnostic criteria for MDD in the *DSM-5*, and suicidal behavior, defined as self-inflicted harm with or without concurrent suicidal ideation, has been reported in up to 50% of patients with MDD; the longer the episodes, the higher the occurrence.^5,6^ Furthermore, suicidal behavior and previous suicide attempts have been shown to increase the risk of death from suicide. Approximately 7% to 13% of patients with nonfatal suicide attempts have been reported to die from suicide at a later time,^7,8^ and up to 7% of patients with MDD who are in contact with specialized psychiatric care have been reported to die from suicide.^6^

However, most patients with MDD are not treated in specialized care,^9,10^ and studies to date have been limited by nonrandom sampling due to nonuniversal access to health care and/or exclusion of primary care data. Thus, it is not established to what extent the aforementioned estimates are representative of patients with MDD as a whole or to what extent suicidal behavior is a risk factor for all-cause mortality. The focus on a specific cause of death (ie, suicide) may underestimate the overall risk of death in the population of patients with MDD and suicidal behavior. Thus, focusing on suicidal behavior and all-cause mortality in a population-wide observational study may result in more clinically relevant and replicable data. In Sweden, all residents have universal access to health care,^11^ with a nominal copayment for health care visits, hospitalizations, and drugs. The opportunities for individual record linkage, together with near-complete information on population health care coverage, including mortality, help to overcome some of the limitations in data that may exist in other countries and from clinical cohorts.

In this study, we investigated the all-cause mortality associated with MDD episodes with suicidal behavior (MDD-SB) compared with MDD episodes without records of suicidal behavior (MDD-non-SB). We also aimed to describe patient clinical characteristics, such as comorbid conditions, treatment patterns, health care resource utilization (HCRU), and work loss. Furthermore, in a separate analysis, we identified risk factors of suicidal behavior in patients with MDD based on information available at the start of an MDD episode.

## Methods

In this population-based cohort study, we used data from the Stockholm MDD Cohort (SMC),^3,4^ which comprises all patients diagnosed with MDD according to *International Classification of Diseases, Tenth Revision* (*ICD-10*) codes F32 to F33 in any health care setting in the region of Stockholm (approximate population, 2.4 million) between 2010 and 2018.^12^ The data in SMC are based on the Regional Healthcare Data Warehouse of Region Stockholm, including information on all individual contacts with health care in Region Stockholm. The information in the data warehouse includes date of death but not cause of death. This project is a part of a framework to facilitate research collaboration between research-based companies and Region Stockholm and registered at the European Network of Centres for Pharmacoepidemiology and Pharmacovigilance (European Union Electronic Register of Post-Authorisation Studies No. 256646). The study was approved by the regional ethics committee, Stockholm, Sweden (No. 2018/546-31), with a waiver for informed consent because all analyses were performed using pseudonymized data. This study followed the Strengthening the Reporting of Observational Studies in Epidemiology (STROBE) reporting guideline.

### Study Cohort and Matching Procedure

We identified all recorded MDD episodes between January 1, 2012, and December 31, 2017, when patients were aged 18 years or older at the start of the episode (MDD baseline) (eAppendix in Supplement 1). Each patient could contribute more than 1 episode. To allow for analyses of MDD baseline conditions, patients residing in Stockholm for 12 months or less before the start of the MDD episode were excluded. We also excluded patients with a history of psychosis, bipolar disorder, manic episode, or dementia. Diagnostic criteria for MDD include suicidal thoughts or attempts (suicidal behavior); therefore, suicidal behavior was defined as *ICD-10* codes for intentional self-harm (X60-X84). Diagnoses of harm of undetermined intent (*ICD-10* codes Y10-Y34) were not included in this study. Records in outpatient or inpatient health care settings were collected, and the date of the first recorded diagnosis of suicidal behavior within an MDD episode was set as the index. At the start of an MDD episode (MDD baseline), patients with MDD and suicidal behavior (MDD-SB group) were compared with all patients with MDD. At index, patients in the MDD-SB group were compared with patient with MDD but without records of suicidal behavior (MDD-non-SB group) by matching each patient in the MDD-SB group (ie, cases) on age (within 2 years), sex, year of MDD diagnosis, and sociodemographic status with up to 5 patients in the MDD-non-SB group (ie, controls). To be eligible for matching, controls were required to have an MDD episode duration at least as long as their matched case’s time from the start of MDD until record of first suicidal behavior. Controls were given the same index date as their matched case (eFigure 1 in Supplement 1). Definitions of inclusion and exclusion criteria are presented in eTable 1 in Supplement 1.

### Statistical Analysis

The data analysis for this study was performed between February 1 and November 1, 2022. All data management and analyses were performed using R, version 3.6.0 software (R Foundation for Statistical Computing). Patient characteristics were described at MDD baseline and index (date of suicidal behavior). In the time-to-event analyses, patients were censored at first instance of emigration from Stockholm, death, a record of an exclusion criterion, or end of follow-up, whichever came first. The time to first suicidal behavior was calculated as the time from MDD baseline until index, and in this analysis, end dates of MDD episodes also qualified as censoring events. To evaluate the association of suicidal behavior with all-cause mortality, we used the Kaplan-Meier method and Cox proportional hazards regression models. Robust SEs were used to account for nonindependence (ie, that 1 patient could have had >1 case of suicidal behavior per MDD episode). Episodes of MDD-SB and MDD-non-SB were followed up from index until the outcome. In a sensitivity analysis, end dates of MDD episodes were also included as censoring events. Departures from the proportional hazards assumption were evaluated using Schoenfeld residuals.

We analyzed psychiatric comorbid conditions, ongoing antidepressant therapy, HCRU, and work loss from 12 months before to 12 months after the index date. Psychiatric comorbid conditions were expressed as cumulative proportions per month; ie, patients were included the first month they were diagnosed with a psychiatric comorbid condition, and this information was carried forward to all later time points. Ongoing treatment with antidepressant therapy (antidepressants, add-on medication, electroconvulsive therapy [ECT], repetitive transcranial magnetic stimulation, and psychotherapy) (eTable 1 in Supplement 1) was calculated as the proportion of patients treated per month. To be defined as receiving ongoing treatment with antidepressant and add-on medication, each patient had to have at least 1 pharmacy dispensation of medication that month or be covered with a medical supply from a previous dispensation. For electroconvulsive therapy, repetitive transcranial magnetic stimulation, or psychotherapy, patients were required to have at least 1 record of a clinical procedure code for that procedure that month. Health care resource utilization was defined as the mean number of outpatient physician visits and inpatient bed days per month, and work loss was defined as the mean number of days not worked per month. Analyses of work loss were performed for patients aged between 20 years and 64 years. Sick leave episodes lasting 14 days or less were not included, as they are not recorded in patient records.

Risk factors for suicidal behavior among patients with MDD were assessed using a study sample restricted to MDD episodes between 2015 and 2017. Only patients residing in Stockholm for 3 years or more prior to the start of the MDD episode were included to ensure a sufficient period of baseline data. Episodes where the start date of the MDD and date of suicidal behavior coincided were excluded. In the model, the outcome of interest was a record of suicidal behavior within 1 year after the start of an MDD episode. Potential risk factors (eTable 2 in Supplement 1) were selected based on previous reports and data availability,^13,14,15,16^ and a Cox proportional hazards regression model was fitted following the steps by Harrell^17^ (details provided in eFigure 6 in Supplement 1). The final prediction model was presented as a nomogram. The importance of each risk factor was measured by partial Wald χ^2^ minus the predictor *df*. We adhered to the Transparent Reporting of a Multivariable Prediction Model for Individual Prognosis or Diagnosis (TRIPOD) statement^18^ in reporting of the results of the prediction model.

## Results

**Figure 1.**
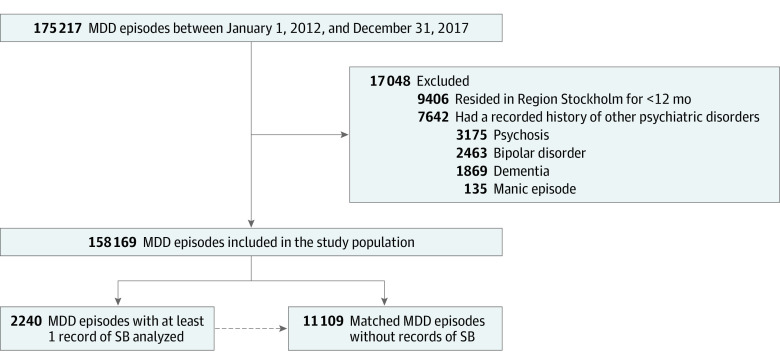
Derivation of the Study Sample Flowchart describing the derivation of the study sample of patients with major depressive disorder (MDD) episodes (as defined in the eAppendix in Supplement 1), including the matched study sample of patients with MDD with and without suicidal behavior (SB). Suicidal behavior was defined using *International Classification of Diseases, Tenth Revision* codes X60 to X84 (intentional self-harm) recorded in any diagnosis position and in both outpatient and inpatient health care settings. The Methods section provides more information. For MDD with SB, the index is the date of first SB within the MDD episode. The matched controls (patients with MDD without SB) are given the same index date as their matched case. The matching procedure is explained in eFigure 1 in Supplement 1.

A total of 158 169 unipolar MDD episodes were identified between January 1, 2012, and December 31, 2017 (Figure 1). Among these episodes, 2240 (1.4%) in 2219 patients had at least 1 record of suicidal behavior (MDD-SB group; mean [SD] patient age, 40.9 [18.6] years; 1415 episodes [63.2%] in women and 825 [36.8%] in men), and 552 (24.6%) of these contained 2 or more records of suicidal behavior. In MDD episodes lasting 5 years or more, 430 of 14 170 (3.0%) had at least 1 suicidal behavior (eFigure 2 in Supplement 1). The most common form of suicidal behavior was intentional self-poisoning (eTable 3 in Supplement 1). The median time from MDD baseline (ie, from start of episode until first suicidal behavior) was 165 days (IQR, 12-510 days). The matched MDD-non-SB control group included 11 109 MDD episodes in 9574 patients (mean [SD] patient age, 40.8 [18.5] years; 7046 episodes [63.4%] in women and 4063 [36.6%] in men) (Figure 1). Patient characteristics at the time of the first suicidal behavior within an MDD episode (index) for the matched study sample are presented in the Table.

**Table.** Patient Characteristics at Time of First Suicidal Behavior Among Episodes of Major Depressive Disorder (MDD) vs Episodes of MDD With No Record of Suicidal Behavior, Matched by Sex, Age, and Socioeconomic Status

Characteristic	No. of episodes (%)	
	MDD episodes with ≥1 record of suicidal behavior^a^^,^^b^	Matched MDD episodes without suicidal behavior^c^
No. of episodes	2240	11 109
No. of patients	2219	9574
Age, mean (SD), y	40.9 (18.6)	40.8 (18.5)
Sex		
Female	1415 (63.2)	7046 (63.4)
Male	825 (36.8)	4063 (36.6)
Psychiatric comorbid conditions^d^		
Anxiety	1288 (57.5)	4612 (41.5)
Stress	865 (38.6)	3153 (28.4)
Sleep disorder	540 (24.1)	1981 (17.8)
Personality disorder	303 (13.5)	335 (3.0)
Substance use disorder	1116 (49.8)	1196 (10.8)
Alcohol use disorder (also included in the substance use disorder category)	722 (32.2)	776 (7.0)
Obsessive compulsive disorder	65 (2.9)	350 (3.2)
Hyperkinetic disorder	256 (11.4)	842 (7.6)
Autism spectrum disorder	93 (4.2)	376 (3.4)
Intentional self-harm (ever)	2240 (100)	158 (1.4)^e^
Event of undetermined intent	111 (5.0)	198 (1.8)
Nonpsychiatric comorbid conditions^d^		
Cardiovascular disorder	204 (9.1)	841 (7.6)
Hypertension	384 (17.1)	1729 (15.6)
Hypothyroidism	128 (5.7)	642 (5.8)
Diabetes, type 1	41 (1.8)	180 (1.6)
Diabetes, type 2	117 (5.2)	494 (4.4)
Inflammatory bowel disease	22 (1.0)	132 (1.2)
Rheumatoid arthritis	17 (0.8)	98 (0.9)
Treatment (history)^f^		
Antidepressants (Anatomical Therapeutic Chemical class N06A)	1880 (83.9)	9012 (81.1)
Add-on medication (lithium, risperidone, olanzapine, aripiprazole, and quetiapine)	399 (17.8)	539 (4.9)
Lithium (also included in the add-on category)	21 (0.9)	25 (0.2)
Psychotherapy	726 (32.4)	3111 (28.0)
Electroconvulsive therapy	75 (3.3)	44 (0.4)
Repetitive transcranial magnetic stimulation	≤5	≤5
Full treatment–resistant depression criteria^g^	270 (12.1)	572 (5.1)
Health care resource utilization, mean (SD)^h^		
Outpatient physician visits	11.4 (9.4)	7.7 (6.9)
Inpatient bed days	11.1 (25.8)	2.1 (8.9)
Work loss, mean (SD), days^i^	87.1 (133.0)	53.0 (107.0)

^a^ Definition of an MDD episode is provided in the eAppendix in Supplement 1.

^b^ Suicidal behavior was defined using *International Classification of Diseases, Tenth Revision* codes X60 to X84 (intentional self-harm) recorded in any diagnosis position and in both outpatient and inpatient health care settings. The Methods section provides more information.

^c^ For MDD with suicidal behavior, index is the date of first suicidal behavior within the MDD episode. The matched controls (MDD without suicidal behavior) are given the same index date as their matched case. The matching procedure is explained in eFigure 1 in Supplement 1.

^d^ Psychiatric and nonpsychiatric comorbid conditions recorded 5 years prior to index. Definitions are provided in eTable 1 in Supplement 1.

^e^ Prior to current MDD episode.

^f^ Treatments recorded at any time prior to index. Definitions are provided in the eAppendix in Supplement 1.

^g^ Treatment-resistant MDD was defined as 3 or more of the treatment trials listed. To qualify as a new treatment trial, an antidepressant or add-on medication had to be initiated within the MDD episode (eAppendix in Supplement 1) and less than 28 days after previous treatment initiation and to have a duration of 28 days or more. Time interval criteria did not apply to electroconvulsive therapy or repetitive transcranial magnetic stimulation.

^h^ Health care resource utilization recorded within 1 year prior to index. The Methods section provides more information.

^i^ Work loss recorded within 1 year prior to index. The Methods section provides more information.

**Figure 2.**
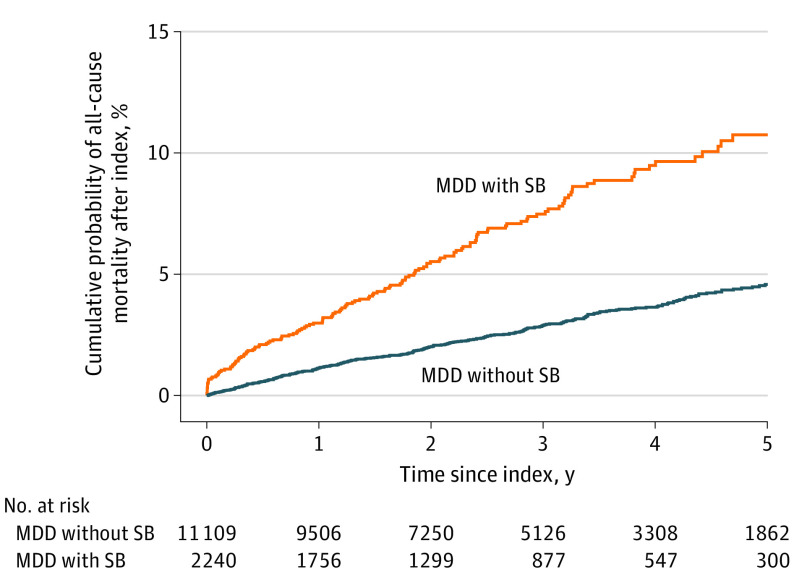
Cumulative Probability of All-Cause Mortality in Patients With Major Depressive Disorder (MDD) With and Without Suicidal Behavior (SB) Definition of an MDD episode is provided in the eAppendix in Supplement 1. Suicidal behavior was defined using *International Classification of Diseases, Tenth Revision* codes X60 to X84 (intentional self-harm) recorded in any diagnosis position and in both outpatient and inpatient health care settings. The Methods section provides more information. For MDD with SB, index is the date of first SB within the MDD episode. The matched controls (MDD without SB) are given the same index date as their matched case. The matching procedure is explained in eFigure 1 in Supplement 1.

The all-cause mortality rate was 2.5 per 100 person-years at risk for the MDD-SB group and 1.0 per 100 person-years at risk for the MDD-non-SB group, based on 466 deaths. This rate corresponds to a hazard ratio of 2.62 (95% CI, 2.15-3.20) (Figure 2). In a sensitivity analysis, MDD episodes were censored at the end of the MDD episode (eFigure 3 in Supplement 1).

Already at the start of the MDD episode, patients in the MDD-SB group appeared different compared with all patients with MDD. For example, they were younger, more often diagnosed while in specialized care, and had higher work loss (eTable 4 in Supplement 1). Patients in the MDD-SB group also had a gradual increase in the prevalence of comorbid conditions from approximately 12 months before index (eFigure 4 in Supplement 1). This increase was most pronounced for anxiety, stress, substance use, and personality disorders. These differences persisted at index between the MDD-SB and MDD-non-SB groups (Table). eFigure 5 in Supplement 1 displays the temporal distribution of monthly HCRU and work loss, with a clear peak at index for the MDD-SB group.

**Figure 3.**
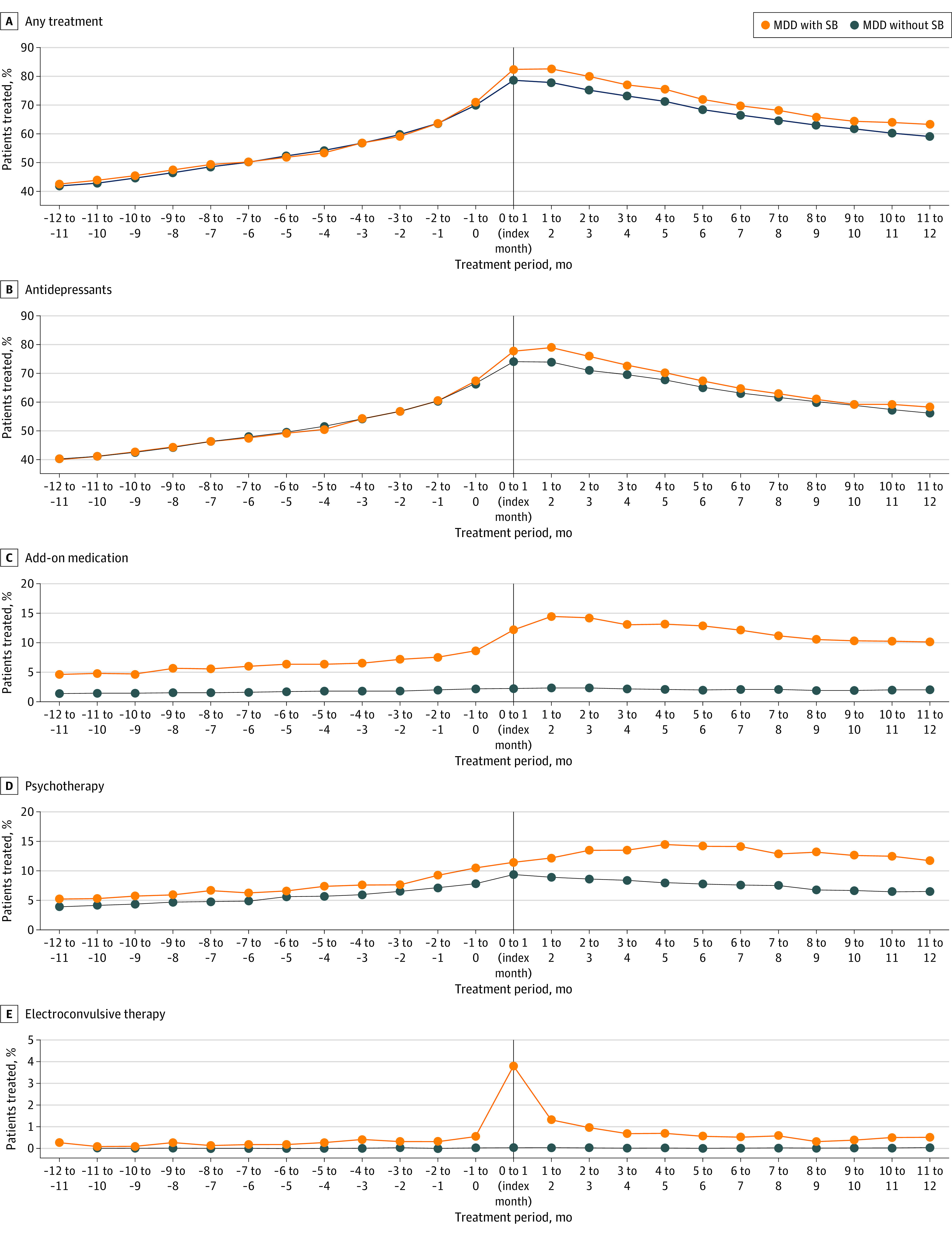
Treatment Categories Ongoing treatment with antidepressant therapy at 12 months before and 12 months after index.

Up until index, 1880 patients (83.9%) in the MDD-SB group and 9012 patients (81.1%) in the MDD-non-SB group were treated with antidepressants (Table). The proportion of patients treated with add-on medication (including lithium in 21 patients [0.9%] and 25 patients [0.2%] in the MDD-SB and MDD-non-SB groups, respectively) and ECT was higher among those in the MDD-SB group both at index and 12 months after compared with the MDD-non-SB group. A total of 82 patients (3.8%) in the MDD-SB group received ECT during the index month, and the provision of psychotherapy ranged between 248 (11.5%) and 275 (14.2%) patients starting at the index month and through the 12 months after the index month (Figure 3).

A risk score for factors associated with suicidal behavior within 1 year after the start of an MDD episode (outcome) was calculated using available variables. The 2 most important risk factors for suicidal behavior were a history of suicidal behavior together with age, which had a U-shaped association with the outcome, with individuals younger than 20 years or 70 years or older having the highest risks. The final risk score also included the following factors (in descending order), the presence of which increased the risk for the outcome: history of substance use, history of sleep disorders, health care level in which MDD was diagnosed, history of antidepressant use, and history of anxiety disorders. eFigure 6 in Supplement 1 displays the final prediction model, presented as a nomogram; all variables evaluated for entry are defined in eTable 2 in Supplement 1. The risk score yielded a C index of 0.78, and the internal bootstrap validation indicated only minimal overfitting. The risk score was well calibrated (eFigure 7 in Supplement 1).

## Discussion

In this cohort study, we analyzed more than 158 000 MDD episodes during 6 years, of which 2240 (1.4%) also included suicidal behavior, with an average time from MDD diagnosis to first record of suicidal behavior of less than 6 months. We found that all-cause mortality was more than doubled in adults with MDD who had a diagnosis of suicidal behavior compared with those with MDD without suicidal behavior, which in and of itself is associated with a doubled risk compared with control patients without MDD.^2,3^ We found an immediate increase in mortality after the first suicidal behavior event, which appeared to be elevated throughout the whole observation period. This suggests that suicidal behavior may be a marker for MDD episodes with an increased risk of mortality; although the SMC data were not linked to information regarding cause of death, and any inference regarding causality remains speculative. However, the incidence of somatic disorders was not increased in the MDD-SB group, indicating that unnatural causes, such as accidents and suicides, may have been the main contributors to the increased mortality.

The proportion of patients with suicidal behavior in our study appears to be lower than in other registry-based studies of patients with MDD^19^ and compared with self-reports from a general population.^5^ A possible explanation could be the virtually full coverage of the patient population with MDD with data from all health care settings in our study, thus not limiting the sample to populations that could be considered to have a higher risk of suicidal behavior, such as patients treated in hospitals or other specialized settings. To reduce the risk of misclassifications, we also chose a stricter definition of suicidal behavior by only including self-harm with intent diagnosed during an ongoing MDD episode. Although previous studies have shown that suicidal behavior is underdiagnosed,^5^ our findings suggest that suicidal behavior in all patients with MDD may not be as common as previously described, although any estimate of suicidal behavior and/or suicide attempt could be subject to uncertainty and methodological considerations. However, in our data, suicidal behavior might be seen as a marker for a subgroup of MDD episodes with more psychiatric comorbidity, higher work loss, and substantially higher all-cause mortality. Thus, developing an evidence-based clinical guideline for both acute and long-term interventions for patients with MDD and suicidal behavior may be warranted.

In our study, the majority of patients were treated with antidepressants at the time of their suicidal behavior, indicating that the treatment may not have been sufficiently effective in ameliorating depressive symptoms and associated suicidal behavior. Although the proportion of patients in the MDD-SB group treated with add-on medications, ECT, and/or psychotherapy increased after the first record of suicidal behavior, the proportions were still low. This finding suggests that treatments for a majority of patients with MDD and suicidal behavior could be improved both before and especially after an suicidal behavior event. Specifically, long-term lithium treatment could be indicated given its suggested association with decreased all-cause mortality in affective disorders.^20^ Still, our findings showed that less than 1% of patients with MDD and suicidal behavior had initiated treatment with lithium at the time of their first recorded suicidal behavior.

The baseline prevalence of nonpsychiatric comorbid conditions was lower in patients with MDD and suicidal behavior compared with all patients with MDD. This finding could be explained by the lower age at baseline, as the differences disappeared after matching. At baseline, patients in the MDD-SB group also had greater proportions of anxiety disorders and substance use than the MDD-non-SB group, which have previously been shown to be factors linked to suicide, suicide attempts, and deliberate self-harm.^13,15,21,22,23,24,25^ Sleep disorders and substance use disorders have previously been reported to be associated with increased all-cause mortality,^26,27^ whereas anxiety has not.^28^ These specific comorbidities were all found to be associated with suicidal behavior in our data. Personality disorders have been linked to suicidal behavior,^13^ and they constituted the highest relative difference in proportions between the MDD-SB group and MDD-non-SB group in our study. In addition, the number of patients with personality disorders almost doubled during the follow-up period, suggesting that they were previously underdiagnosed. In absolute numbers, however, the majority of suicidal behavior events were not linked to personality disorders.

The frequency of health care contacts and high rates of psychiatric comorbidity among patients with MDD who later develop suicidal behavior presents important opportunities for health care to identify and treat these patients at an early stage. A comprehensive approach that also includes diagnosing and addressing these comorbidities, in addition to treating depressive symptoms, would be beneficial. Should evidence-based guidelines be developed on how to best optimize treatments to avoid suicidal behavior, they should include early detection of potential patients with MDD and suicidal behavior. With future evidence-based guidelines in mind, we calculated a risk score based on variables present in our data. The risk score could be explored as a tool to help identify the risk of suicidal behavior among patients with MDD in clinical practice. In the risk score, the most important factors associated with suicidal behavior within 1 year after the start of an MDD episode were history of suicidal behavior and age, followed by history of substance use and sleep disorders. However, it is important to emphasize that the predictive ability of this tool needs to be evaluated in other patient populations and to acknowledge that some important variables, such as family history of psychiatric conditions, were not included.^23^ Thus, this tool should be used as a complement and should not supersede a thorough clinical evaluation. It is important to acknowledge that not all patients with suicidal behavior present for treatment,^29^ even in a universal health care system, which limits the assessment of risk factors and possibilities for prevention.

### Limitations

This study had several limitations. We defined suicidal behavior as any diagnosis of intentional self-harm (*ICD-10* codes X60-X84) and did not include diagnoses of harm of undetermined intent (*ICD-10* codes Y10-Y34). Even so, this broad definition probably gives a comprehensive picture of suicidal behavior among patients with MDD. An suicidal behavior episode was defined by records of consecutive events, and any additional records of suicidal behavior at later time points were attributed to a new episode. This strict definition might have slightly overestimated the number of suicidal behavior episodes, which should be contrasted to previous reports that suicidal behavior is underdiagnosed.^5^ The study population consisted of unipolar MDD episodes and excluded patients with records of bipolar disorder, dementia, or psychosis before or at baseline, and patients who developed any of these conditions later were censored in time-to-event analyses, limiting our ability to draw conclusions on MDD-SB in these patient groups. However, suicidal behavior may also be associated with an increased risk of mortality in other psychiatric disorders. Other comorbid conditions, such as anxiety, substance use, and personality disorders, were included, which is important since they are common among patients with MDD in general, as well as among patients with MDD and suicidal behavior in particular.

## Conclusions

In this cohort study, we found that 1.4% of MDD episodes in Stockholm, Sweden, involved records of suicidal behavior, which is lower than previously reported.^6,7,8^ Among these patients, the all-cause mortality was more than doubled compared with MDD episodes without records of suicidal behavior. Our results also indicate that patients at risk for suicidal behavior can be identified at an early stage to allow for enhanced monitoring and optimized treatment with the goal of preventing suicidal behavior and reducing mortality.

## Notes

Supplement 1. **eAppendix.** Definition of Major Depressive Disorder (MDD) Episode **eFigure 1.** Matching Procedure for Patients With Major Depressive Disorder (MDD) With and Without Suicidal Behavior (SB) **eTable 1.** Definitions of Inclusion and Exclusion Criteria, Treatments, and Comorbid Conditions **eTable 2.** Definition of Variables Included in the Development of the Cox Proportional Hazards Model on Determinants for Suicidal Behavior (SB) Within 1 Year After Start of a Major Depressive Disorder (MDD) Episode **eFigure 2.** Cumulative Proportion of Major Depressive Disorder (MDD) Episodes With Records of Suicidal Behavior (SB) After Start of an MDD Episode, by Age Strata **eTable 3.** Specification of Type of Suicidal Behavior (SB) Events in Descending Order Among the 2,240 Patients With Major Depressive Disorder (MDD) and SB **eFigure 3.** Cumulative Probability of All-Cause Mortality in Patients With Major Depressive Disorder (MDD) With and Without Suicidal Behavior (MDD-SB) Compared With MDD-Non-SB, Sensitivity Analysis **eTable 4.** Characteristics at Start of the Major Depressive Disorder (MDD) Episodes (MDD-Baseline) Among Patients With Records of Suicidal Behavior (SB) Within the Current MDD Episode Compared With All MDD Episodes (With and Without Records of SB) **eFigure 4.** Prevalence of Psychiatric Comorbid Conditions 12 Months Before and 12 Months After Index (Time of First Suicidal Behavior [SB] Within the Major Depressive Disorder [MDD] Episode) **eFigure 5.** Mean Monthly Health Care Resource Utilization (HCRU) and Work Loss 12 Months Before and 12 Months After Index (Time of First Suicidal Behavior [SB] Within the Major Depressive Disorder [MDD] Episode) **eFigure 6.** Nonogram for the Cox Proportional Hazards Model on Determinants for Suicidal Behavior Within 1 Year After Start of a Major Depressive Disorder (MDD) Episode, Based on Patients With MDD Episodes Between 2015 and 2017 Residing in Stockholm for at Least 3 Year Prior to Start of MDD **eFigure 7.** Calibration of the Cox Proportional Hazards Model on Determinants for Suicidal Behavior (SB) Within 1 Year After Start of a Major Depressive Disorder (MDD) Episode

Supplement 2.
Data Sharing Statement
