# Supplementary material for: Determinants and Outcomes of Suicidal Behavior Among Patients With Major Depressive Disorder
Source: JAMA Psychiatry. 2023 Aug 16;80(12):1218–25. doi: 10.1001/jamapsychiatry.2023.2833 (PMC10433143; doi:10.1001/jamapsychiatry.2023.2833)
Supplement: Supplement 1. — eAppendix. Definition of Major Depressive Disorder (MDD) Episode eFigure 1. Matching Procedure for Patients With Major Depressive Disorder (MDD) With and Without Suicidal Behavior (SB) eTable 1. Definitions of Inclusion and Exclusion Criteria, Treatments, and Comorbid Conditions eTable 2. Definition of Variables Included in the Development of the Cox Proportional Hazards Model on Determinants for Suicidal Behavior (SB) Within 1 Year After Start of a Major Depressive Disorder (MDD) Episode eFigure 2. Cumulative Proportion of Major Depressive Disorder (MDD) Episodes With Records of Suicidal Behavior (SB) After Start of an MDD Episode, by Age Strata eTable 3. Specification of Type of Suicidal Behavior (SB) Events in Descending Order Among the 2,240 Patients With Major Depressive Disorder (MDD) and SB eFigure 3. Cumulative Probability of All-Cause Mortality in Patients With Major Depressive Disorder (MDD) With and Without Suicidal Behavior (MDD-SB) Compared With MDD-Non-SB, Sensitivity Analysis eTable 4. Characteristics at Start of the Major Depressive Disorder (MDD) Episodes (MDD-Baseline) Among Patients With Records of Suicidal Behavior (SB) Within the Current MDD Episode Compared With All MDD Episodes (With and Without Records of SB) eFigure 4. Prevalence of Psychiatric Comorbid Conditions 12 Months Before and 12 Months After Index (Time of First Suicidal Behavior [SB] Within the Major Depressive Disorder [MDD] Episode) eFigure 5. Mean Monthly Health Care Resource Utilization (HCRU) and Work Loss 12 Months Before and 12 Months After Index (Time of First Suicidal Behavior [SB] Within the Major Depressive Disorder [MDD] Episode) eFigure 6. Nonogram for the Cox Proportional Hazards Model on Determinants for Suicidal Behavior Within 1 Year After Start of a Major Depressive Disorder (MDD) Episode, Based on Patients With MDD Episodes Between 2015 and 2017 Residing in Stockholm for at Least 3 Year Prior to Start of MDD eFigure 7. Calibration of the Cox Proportional Hazards [file jamapsychiatry-e232833-s001.pdf]

Supplemental Online Content

Lundberg J, Cars T, Lampa E, et al. Determinants and outcomes of suicidal behavior among patients with major depressive disorder. *JAMA Psychiat*. Published online August 16, 2023. doi:10.1001/jamapsychiatry.2023.2833

eAppendix. Definition of Major Depressive Disorder (MDD) Episode

eFigure 1. Matching Procedure for Patients With Major Depressive Disorder (MDD) With and Without Suicidal Behavior (SB)

eTable 1. Definitions of Inclusion and Exclusion Criteria, Treatments, and Comorbid Conditions

eTable 2. Definition of Variables Included in the Development of the Cox Proportional Hazards Model on Determinants for Suicidal Behavior (SB) Within 1 Year After Start of a Major Depressive Disorder (MDD) Episode

eFigure 2. Cumulative Proportion of Major Depressive Disorder (MDD) Episodes With Records of Suicidal Behavior (SB) After Start of an MDD Episode, by Age Strata

eTable 3. Specification of Type of Suicidal Behavior (SB) Events in Descending Order Among the 2,240 Patients With Major Depressive Disorder (MDD) and SB

eFigure 3. Cumulative Probability of All-Cause Mortality in Patients With Major Depressive Disorder (MDD) With and Without Suicidal Behavior (MDD-SB) Compared With MDD-Non-SB, Sensitivity Analysis

eTable 4. Characteristics at Start of the Major Depressive Disorder (MDD) Episodes (MDD-Baseline) Among Patients With Records of Suicidal Behavior (SB) Within the Current MDD Episode Compared With All MDD Episodes (With and Without Records of SB)

eFigure 4. Prevalence of Psychiatric Comorbid Conditions 12 Months Before and 12 Months After Index (Time of First Suicidal Behavior [SB] Within the Major Depressive Disorder [MDD] Episode)

eFigure 5. Mean Monthly Health Care Resource Utilization (HCRU) and Work Loss 12 Months Before and 12 Months After Index (Time of First Suicidal Behavior [SB] Within the Major Depressive Disorder [MDD] Episode)

eFigure 6. Nonogram for the Cox Proportional Hazards Model on Determinants for Suicidal Behavior Within 1 Year After Start of a Major Depressive Disorder (MDD) Episode, Based on Patients With MDD Episodes Between 2015 and 2017 Residing in Stockholm for at Least 3 Years Prior to Start of MDD

eFigure 7. Calibration of the Cox Proportional Hazards Model on Determinants for Suicidal Behavior (SB) Within 1 Year After Start of a Major Depressive Disorder (MDD) Episode

This supplemental material has been provided by the authors to give readers additional information about their work.

© 2023 Lundberg J et al. *JAMA Psychiatry.*

eAppendix. Definition of Major Depressive Disorder (MDD) Episode

Since we do not have clinical depression ratings scales recorded at regular timepoints for all patients, we invented a definition in order to get a proxy for the length of an MDD-episode (or rather, the duration of healthcare contacts related to MDD). This was operationally defined based on recorded activities related to depression:

(1) We identified each patient's first recorded MDD-diagnosis (ICD10: F32-Depressive episode and F33-Recurrent depressive episode), which marked the start of the first documented depressive episode (i.e., the MDD-baseline).

(2) For each patient we further analyzed the time from first recorded MDD-diagnosis to subsequent depressive events. If the time-interval between the two records of depressive events was ≤365 days the episode was categorized as ongoing, while if the time interval was >365 days the episode was categorized as closed at the date of the last depressive event. Depressive events were defined as: (1) records of depression diagnoses, (2) filled prescriptions of antidepressants (AD; ATC: N06A) and add-on medication for depression (lithium, risperidone, olanzapine, aripiprazole, and quetiapine (>100 mg)), electroconvulsive therapy (ECT), repetitive transcranial magnetic stimulation (rTMS), or treatment with psychotherapy. If the last depressive event was a dispensation of either AD or add-on medication, we extended the episode with the number of dispensed tablets (i.e., a maximum of 100 days was added).

(3) When an episode is categorized as closed, a subsequent record of a depression diagnosis code would mark the start of a new episode.

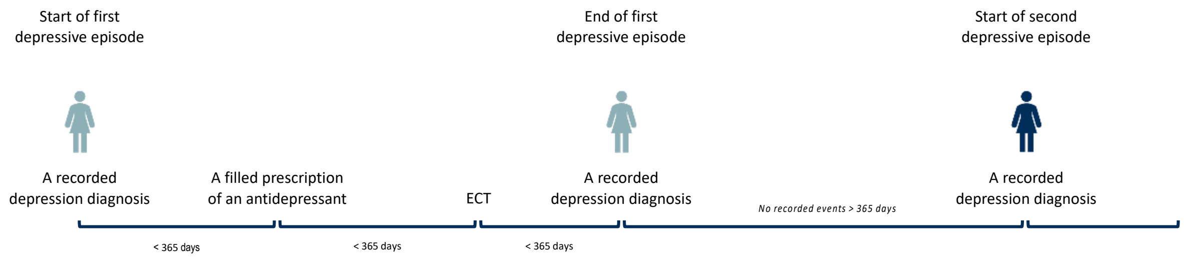

© 2023 Lundberg J et al. *JAMA Psychiatry.*

eFigure 1. Matching Procedure for Patients With Major Depressive Disorder (MDD)[1] With and Without Suicidal Behavior (SB)[2]

Each major depressive disorder (MDD) episode[a] with at least one record of suicidal behavior (SB)[b] (MDD-SB) within the episode was matched to an MDD-episode without a record of SB (MDD-non-SB). The episodes were matched by patients' age, sex, and socioeconomic status. The matched MDD-episodes in MDD-non-SB group also had to have an MDD-duration at least as long as the time from start of MDD until record of first SB for the episodes to which they were matched within the MDD-SB group. Matched controls were given the same index date as the matched case (see figure below). This means that if an MDD-episode has a record of SB 100 days after start of the MDD-episode, the index date was start of the MDD-episode + 100 days. The five matched MDD-episodes (with no records of SB) were given the same index, i.e., start of depressive episode + 100 days.

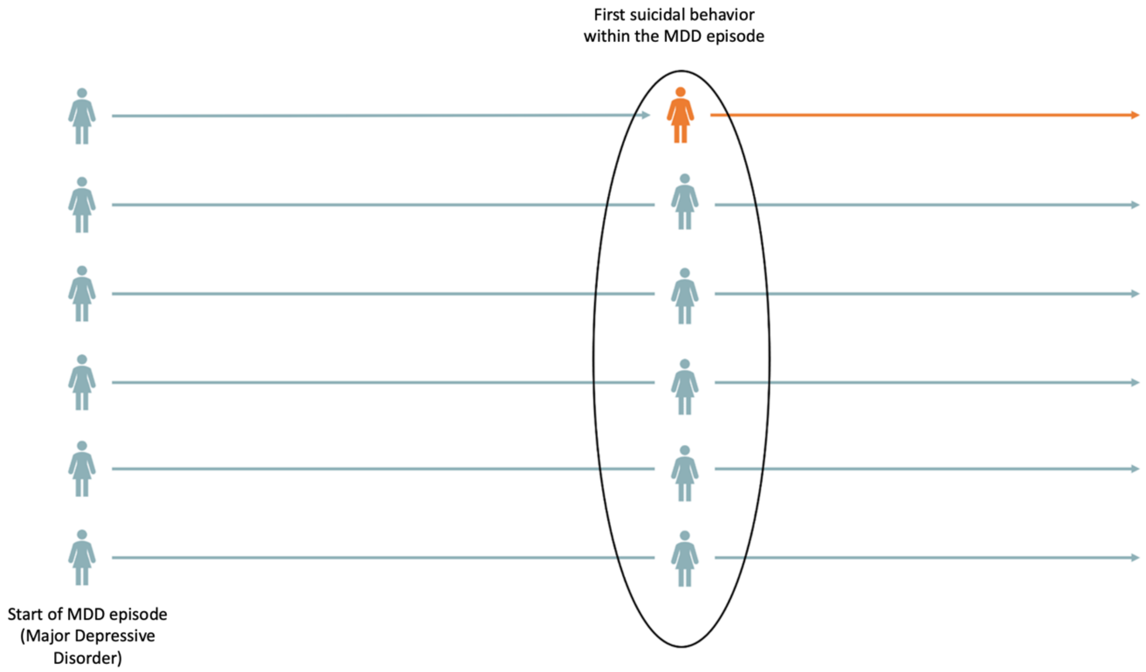

[a] Definition of a major depressive disorder (MDD) episode is displayed in Supplement A.

[b] Suicidal behavior (SB) was defined by the ICD10 codes X60-X84 (intentional self-harm) recorded in any diagnosis position and in both outpatient and inpatient healthcare settings. See methods section for more information.

© 2023 Lundberg J et al. *JAMA Psychiatry.*

eTable 1. Definitions of inclusion and exclusion criteria, treatments, and comorbid conditions

Condition	Included codes
**Psychiatric conditions**	
MDD diagnosis	ICD10:<br>F32 – Depressive episode<br>F33 – Recurrent depressive disorder
Psychosis	ICD10:<br>F20-F29 Schizophrenia, schizotypal and delusional disorders
Manic episode	ICD10:<br>F30 Manic episode
Bipolar disorders	ICD10:<br>F31 Bipolar affective disorder
Dementia	ICD10:<br>F00 – Dementia in Alzheimer disease<br>F01 – Vascular dementia<br>F02 – Dementia in other diseases classified elsewhere<br>F03 – Unspecified dementia
Anxiety	ICD10:<br>F41– Other anxiety disorders
OCD	ICD10:<br>F42– Obsessive compulsive disorder
Stress	ICD10:<br>F43 – Reaction to severe stress, and adjustment disorders
Substance use	ICD10:<br>F10-F19 – Mental and behavioral disorders due to psychoactive substance use
Sleep disorders	ICD10:<br>G47 – Sleep disorders<br>F51 – Nonorganic sleep disorders
Personality disorders	ICD10:<br>F60 – Specific personality disorders
Hyperkinetic disorders	ICD10:<br>F90 – Hyperkinetic disorders
ASD – Autism spectrum disorders	ICD10:<br>F840 – Childhood autism<br>F841 – Atypical autism<br>F845 – Asperger syndrome
Intentional self-harm	ATC:<br>X60-X84 – Intentional self-harm
**Non-psychiatric conditions**	
Cardiovascular disease	ICD10:<br>I20-I25, I50, I110, I42 (excl I42.1 and I42.2), I43, I48, I60-I64, G45, I70-I72, I73.1, I73.9, I74, I77.3, I77.6, I77.8, I79
Hypertension	ICD10:<br>I10-I15
Diabetes mellitus type II	ICD10:<br>E11 – Diabetes mellitus type II
Rheumatoid arthritis	ICD10:<br>M05 – Seropositive rheumatoid arthritis<br>M06 – Other rheumatoid arthritis
Inflammatory bowel disease	ICD10:<br>K50 – Crohn disease [regional enteritis]<br>K51 – Ulcerative colitis
Hypothyroidism	ICD10:<br>E03 – Other hypothyroidism
**Antidepressant therapy**	
AD	ATC:<br>N06A – Antidepressants
Add-on medication	ATC:

© 2023 Lundberg J et al. *JAMA Psychiatry.*

	N05AN01 – Lithium<br>N05AX08 – Risperidone<br>N05AH03 – Olanzapine<br>N05AX12 – Aripiprazole<br>N05AH04 – Quetiapine (>100 mg)
ECT	Clinical procedure codes:<br>DA006 – ECT, unspecified<br>DA024 – ECT, unilateral<br>DA025 – ECT, bilateral
rTMS	Clinical procedure codes:<br>DU050 – Repetitive Transcranial Magnetic Stimulation
Psychotherapy	Clinical procedure codes:<br>DU008 – Psychodynamic therapy<br>DU009 – Psychotherapy, other<br>DU010 – Psychotherapy, cognitive<br>DU011 – Psychotherapy, CBT<br>DU013 – Psychotherapy, MBT<br>DU020 – Psychotherapy, systemic<br>DU021 – Psychotherapy, DBT<br>DU022 – Psychotherapy, IPT<br>DU023 – Psychopedagogical

© 2023 Lundberg J et al. *JAMA Psychiatry.*

eTable 2. Definition of Variables Included in the Development of the Cox Proportional Hazards Model on Determinants for Suicidal Behavior (SB)[a] Within 1 Year After Start of a Major Depressive Disorder (MDD) Episode[b]

Variable	Definition
Age	Age at start of the MDD-episode
Sex	Men/Women
Healthcare level	This variable presents the healthcare level where the first MDD-diagnosis within the current MDD-episode was recorded.<br>Two levels: (1) Non-psychiatric care and (2) psychiatry care. The level non-psychiatric mainly comprise patients diagnosed in primary health care settings.
Pharmacological treatment for depression	This binary variable (yes/no) presents if the patient has been treated with pharmacological treatment (AD and add-on medication, see supplement C) for depression within three year before start of current MDD-episode.
Treatment with ECT	This binary variable (yes/no) presents if the patient within three years before start of current MDD episode) has been treated with either ECT
Treatment with psychotherapy	This binary variable (yes/no) presents if the patient within three years before index (start of current MDD episode) has been treated with psychotherapy.
**Psychiatric comorbid conditions**	
Sleep disorders	This binary variable indicates if the patient within the past three years before start of current MDD-episode either has been diagnosed for sleep disorders (ICD10:G47 or F51) or at least one filled prescription of a sedatives (ATC: N05C)
Anxiety	This binary variable indicates if the patient within the past three years before start of current MDD-episode either has been diagnosed for anxiety (ICD10:F41)
Substance use	This binary variable indicates if the patient within the past three years before start of current MDD-episode either has been diagnosed with mental and behavioral disorders due to psychoactive substance use (ICD10:F10-F19).
Personality disorder	This binary variable indicates if the patient within the past three years before start of current MDD-episode been diagnosed with personality disorder (ICD10:F60)
Hyperkinetic disorders	This binary variable indicates if the patient within the past three years before start of current MDD-episode been diagnosed for hyperkinetic disorders (ICD10:F90)
Autism spectrum disorder - ASD	This binary variable indicates if the patient within the past three years before start of current MDD-episode been diagnosed with autism spectrum disorder (ICD10:F840, F841, F845)
Intentional self-harm	This binary variable indicates if the patient within the past three years before start of current MDD-episode has a recording of intentional self-harm (ICD10:X60-X84). We included diagnosis of intentional self-harm recorded both in outpatient and inpatient care.
**Work-loss**	
Lost workdays	This continuous variable includes the number of lost workdays during the past 12 months before start of current MDD-episode. We included both sick-leave days and days with disability pension.

[a] Suicidal behavior (SB) was defined by the ICD10 codes X60-X84 (intentional self-harm) recorded in any diagnosis position and in both outpatient and inpatient healthcare settings. See methods section for more information.

[b] Definition of a major depressive disorder (MDD) episode is displayed in Supplement A.

eFigure 2. Cumulative Proportion of Major Depressive Disorder (MDD) Episodes[a] With Records of Suicidal Behavior (SB)[b] After Start of an MDD Episode, by Age Strata

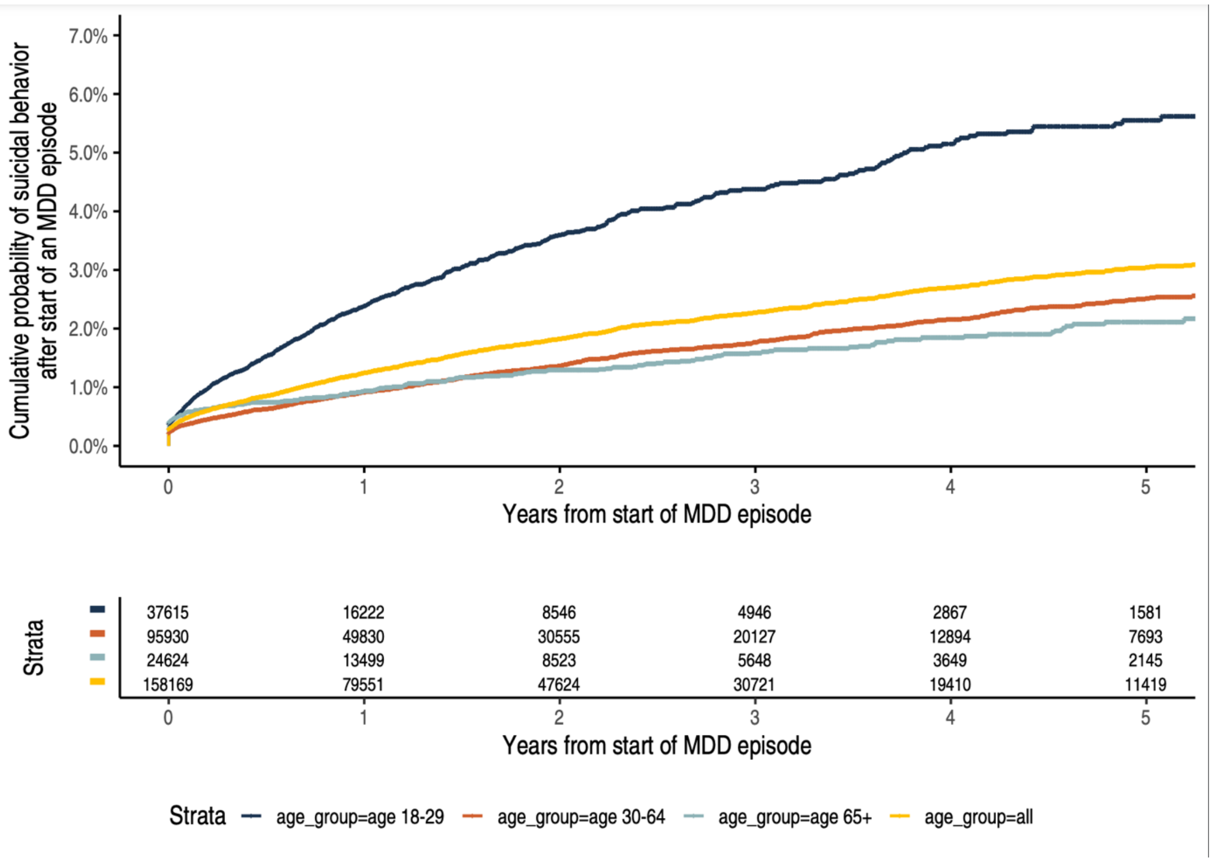

[a] Definition of a major depressive disorder (MDD) episode is displayed in Supplement A.

[b] Suicidal behavior (SB) was defined by the ICD10 codes X60-X84 (intentional self-harm) recorded in any diagnosis position and in both outpatient and inpatient healthcare settings. See methods section for more information.

© 2023 Lundberg J et al. *JAMA Psychiatry.*

eTable 3. Specification of Type of Suicidal Behavior (SB) Events[a] in Descending Order Among the 2,240 Patients With Major Depressive Disorder (MDD)[b] and SB. Note that patients may have had more than one type of SB-event.

	ICD10-code	N (%)
Intentional self-poisoning by and exposure to other and unspecified drugs, medicaments, and biological substances	X64	1,538 (68.6%)
Intentional self-harm by sharp object	X78	314 (14.0%)
Intentional self-poisoning by and exposure to antiepileptic, sedative-hypnotic, antiparkinsonism, and psychotropic drugs, not elsewhere classified	X61	116 (5.2%)
Intentional self-poisoning by and exposure to nonopioid analgesics, antipyretics, and antirheumatics	X60	66 (2.9%)
Intentional self-poisoning by and exposure to alcohol	X65	45 (2.0%)
Intentional self-harm by hanging, strangulation, and suffocation	X70	44 (2.0%)
Intentional self-harm by jumping from a high place	X80	43 (1.9%)
Intentional self-harm by other specified means	X83	30 (1.3%)
Intentional self-poisoning by and exposure to other and unspecified chemicals and noxious substances	X69	28 (1.3%)
Intentional self-harm by unspecified means	X84	17 (0.8%)
Intentional self-poisoning by and exposure to narcotics and psychodysleptics [hallucinogens], not elsewhere classified	X62	15 (0.7%)
Intentional self-poisoning by and exposure to other drugs acting on the autonomic nervous system	X63	10 (0.4%)
Intentional self-harm by crashing of motor vehicle	X82	10 (0.4%)
Intentional self-harm by drowning and submersion	X71	8 (0.4%)
Intentional self-poisoning by and exposure to carbon monoxide and other gases and vapors	X67	7 (0.3%)
Intentional self-harm by smoke, fire, and flames	X76	6 (0.3%)
Intentional self-harm by jumping or lying before moving object	X81	6 (0.3%)
Intentional self-poisoning by and exposure to organic solvents and halogenated hydrocarbons and their vapors	X66	<5
Intentional self-poisoning by and exposure to pesticides	X68	<5
Intentional self-harm by rifle, shotgun and larger firearm discharge	X73	<5
Intentional self-harm by other and unspecified firearm discharge	X74	<5
Intentional self-harm by blunt object	X79	<5

[a] Suicidal behavior (SB) was defined by the ICD10 codes X60-X84 (intentional self-harm) recorded in any diagnosis position and in both outpatient and inpatient healthcare settings. See methods section for more information.

[b] Definition of a major depressive disorder (MDD) episode is displayed in Supplement A.

© 2023 Lundberg J et al. *JAMA Psychiatry.*

eFigure 3. Cumulative Probability of All-Cause Mortality in Patients With Major Depressive Disorder (MDD)[a], With and Without Suicidal Behavior (MDD-SB)[b] Compared With MDD-Non-SB,[c] Sensitivity Analysis

In this sensitivity analysis, MDD-episodes were censored at the end of the MDD-episode.

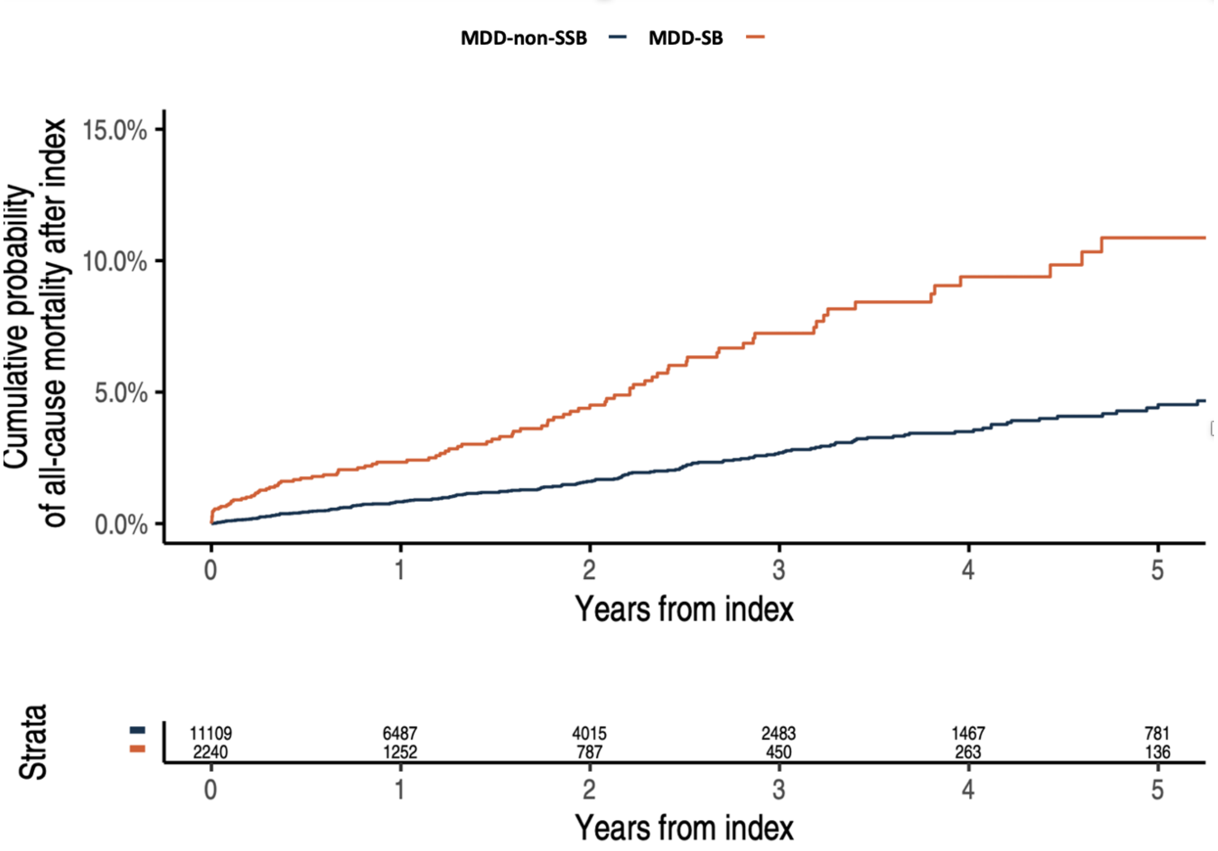

[a] Definition of a major depressive disorder (MDD) episode is displayed in Supplement A.

[b] Suicidal behavior (SB) was defined by the ICD10 codes X60-X84 (intentional self-harm) recorded in any diagnosis position and in both outpatient and inpatient healthcare settings. See methods section for more information.

[c] For MDD-SB, index is the date of first SB within the MDD-episode. The matched controls (MDD-non-SB) are given the same index date as their matched case. The matching procedure is explained in Supplement B.

© 2023 Lundberg J et al. *JAMA Psychiatry.*

eTable 4. Characteristics at Start of the Major Depressive Disorder (MDD) Episodes (MDD-Baseline) Among Patients With Records of Suicidal Behavior (SB) Within the Current MDD Episode Compared With All MDD Episodes (With and Without Records of SB)

	MDD-episodes[a] with SB[b]	All MDD-episodes[a]
N (episodes)	2,240	158,169
N patients	2,219	145,577
Age (mean, SD)	40.0 (18.5)	44.8 (18.0)
Female sex (%)	1,415 (63.2%)	102,310 (64.7%)
**Healthcare level at start of MDD episode**		
Psychiatric care	1,104 (49.3%)	38,375 (24.3%)
Non psychiatric care	1,136 (50.7%)	119,794 (75.7%)
**Psychiatric comorbid conditions[c]**		
Anxiety	1,005 (44.9%)	48,141 (30.4%)
Stress	682 (30.4%)	41,426 (26.2%)
Sleep disorders	477 (21.3%)	27,493 (17.4%)
Personality disorders	193 (8.6%)	2,809 (1.8%)
Substance use disorder	738 (32.9%)	14,548 (9.2%)
Alcohol use disorder	525 (23.4%)	9,544 (6.0%)
Obsessive compulsive disorders	41 (1.8%)	2,568 (1.6%)
Hyperkinetic disorders	175 (7.8%)	6,379 (4.0%)
Autism Spectrum Disorder	66 (2.9%)	2,031 (1.3%)
Intentional self-harm	759 (33.9%)	2,645 (1.7%)
Event of undetermined intent	83 (3.7%)	2,370 (1.5%)
**Non-psychiatric comorbid conditions[c]**		
Cardiovascular disorders	178 (7.9%)	13,804 (8.7%)
Hypertension	337 (15.0%)	27,707 (17.5%)
Hypothyroidism	112 (5.0%)	10,487 (6.6%)
Diabetes mellitus, Type 1	42 (1.9%)	2,456 (1.6%)
Diabetes mellitus, Type 2	104 (4.6%)	7,577 (4.8%)
Inflammatory bowel disease	19 (0.8%)	1,683 (1.1%)
Reumathoid arthritis	17 (0.8%)	1,284 (0.8%)
**Treatment history[d]**		
Antidepressants (ATC: N06A)	1,268 (56.6%)	69,507 (43.9%)
Add-on medication	183 (8.2%)	3,052 (1.9%)
Lithium (also included in the add-on category)	<=5	97 (0.1%)
Psychotherapy	325 (14.5%)	18,464 (11.7%)
Electroconvulsive therapy (ECT)	21 (0.9%)	160 (0.1%)
Repetitive transcranial magnetic stimulation	<=5	<=5
**Healthcare resource utilization (HCRU)[e]**		
Outpatient physician visits (Mean, SD)	8.1 (8.6)	6.2 (7.1)
Inpatient bed-days (Mean, SD)	5.5 (18.5)	1.8 (8.2)
**Work-loss (days)[f]**		
Mean, SD	53.2 (111.9)	34.2 (87.7)

[a] Definition of a major depressive disorder (MDD) episode is displayed in Supplement A.
[b] Suicidal behavior (SB) was defined by the ICD10 codes X60-X84 (intentional self-harm) recorded in any diagnosis position and in both outpatient and inpatient healthcare settings. See methods section for more information.
[c] Psychiatric and non-psychiatric comorbid conditions recorded five years prior to the start of the MDD-episode. Definitions are displayed in Supplement C.
[d] Treatments recorded at any time prior to the start of the MDD-episode. Definitions are displayed in Supplement C.
[e] HCRU recorded within one year prior to the start of the MDD-episode. See methods section for more information.
[f] Work-loss recorded within one year prior to the start of the MDD-episode. See methods section for more information.

© 2023 Lundberg J et al. *JAMA Psychiatry.*

eFigure 4. Prevalence of Psychiatric Comorbid Conditions[a] 12 Months Before and 12 Months After Index (Time of First Suicidal Behavior [SB][b] Within the Major Depressive Disorder [MDD] Episode[c])

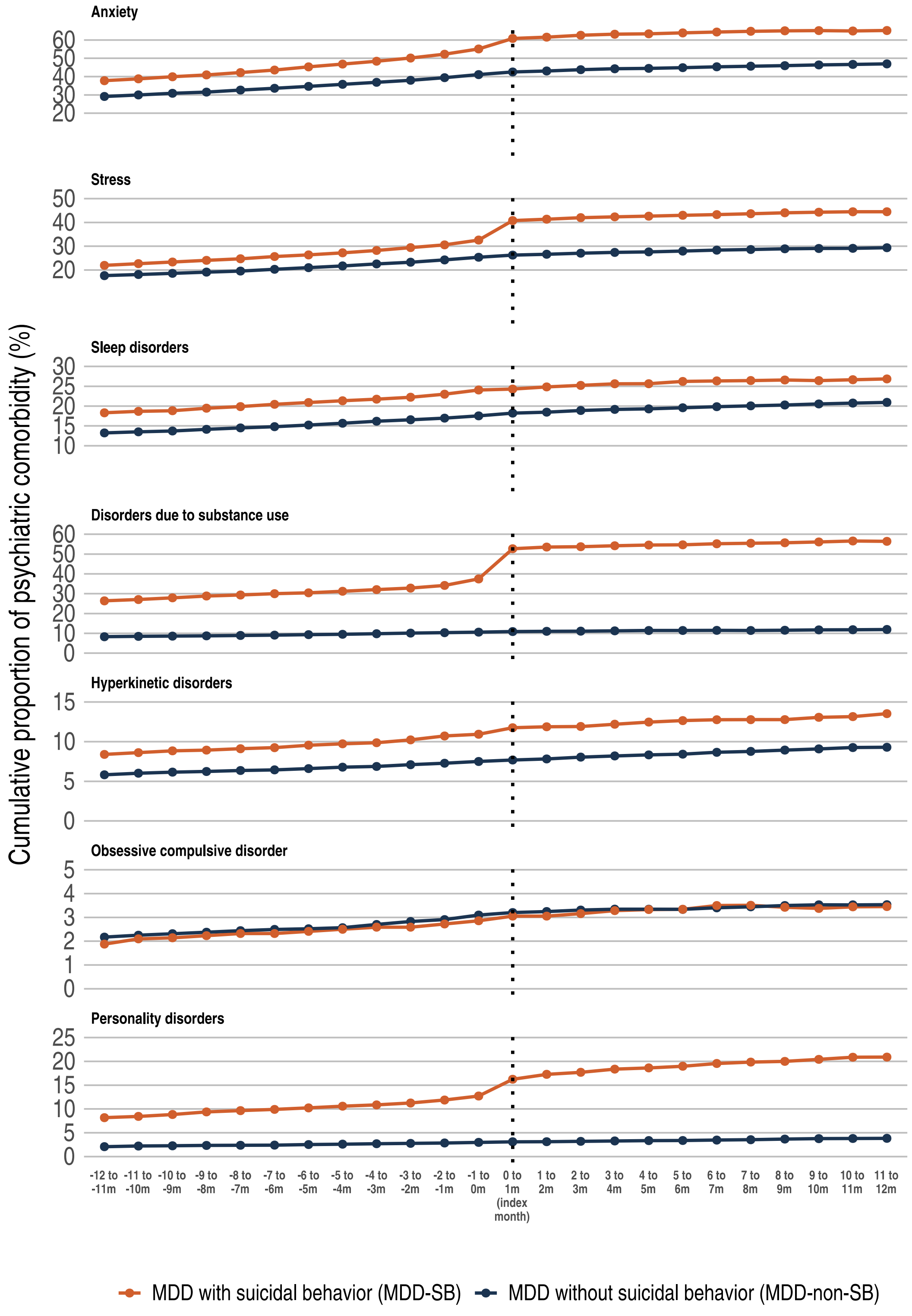

[a] Psychiatric comorbid conditions are defined in Supplement C.
[b] Suicidal behavior (SB) was defined by the ICD10 codes X60-X84 (intentional self-harm) recorded in any diagnosis position and in both outpatient and inpatient healthcare settings. See methods section for more information.
[c] Definition of a major depressive disorder (MDD) episode is displayed in Supplement A.

© 2023 Lundberg J et al. *JAMA Psychiatry.*

eFigure 5. Mean Monthly Health Care Resource Utilization (HCRU) and Work Loss 12 Months Before and 12 Months After Index (Time of First Suicidal Behavior [SB][a] Within the Major Depressive Disorder [MDD] Episode[b])

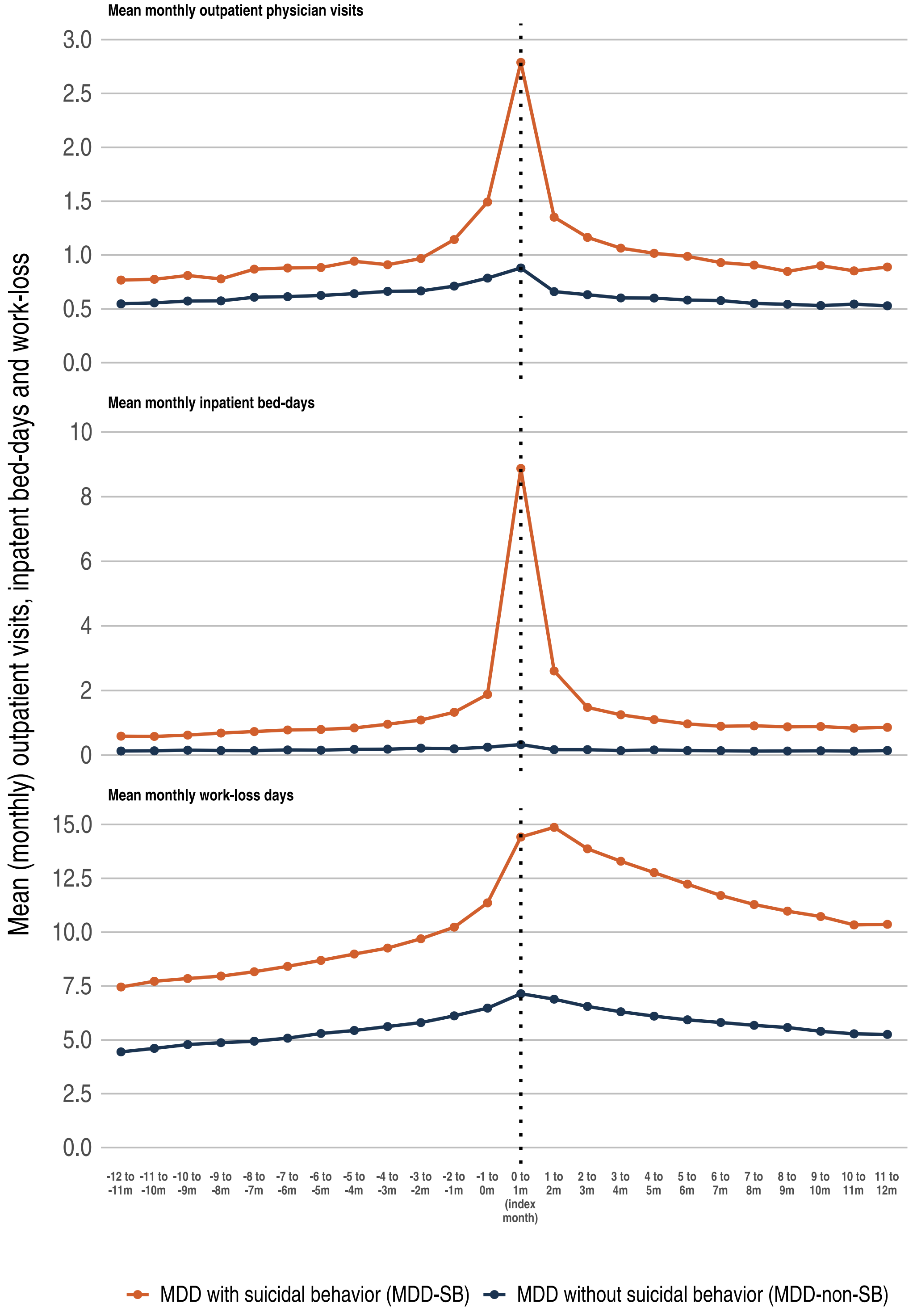

[a] Suicidal behavior (SB) was defined by the ICD10 codes X60-X84 (intentional self-harm) recorded in any diagnosis position and in both outpatient and inpatient healthcare settings. See methods section for more information.

[b] Definition of a major depressive disorder (MDD) episode is displayed in Supplement A.

© 2023 Lundberg J et al. *JAMA Psychiatry.*

eFigure 6. Nomogram[a,b] for the Cox Proportional Hazards Model on Determinants for Suicidal Behavior (SB)[c] Within 1 Year After Start of a Major Depressive Disorder (MDD) Episode[d], Based on Patients With MDD Episodes Between 2015 and 2017 Residing in Stockholm for at Least 3 Years Prior to Start of MDD[e]

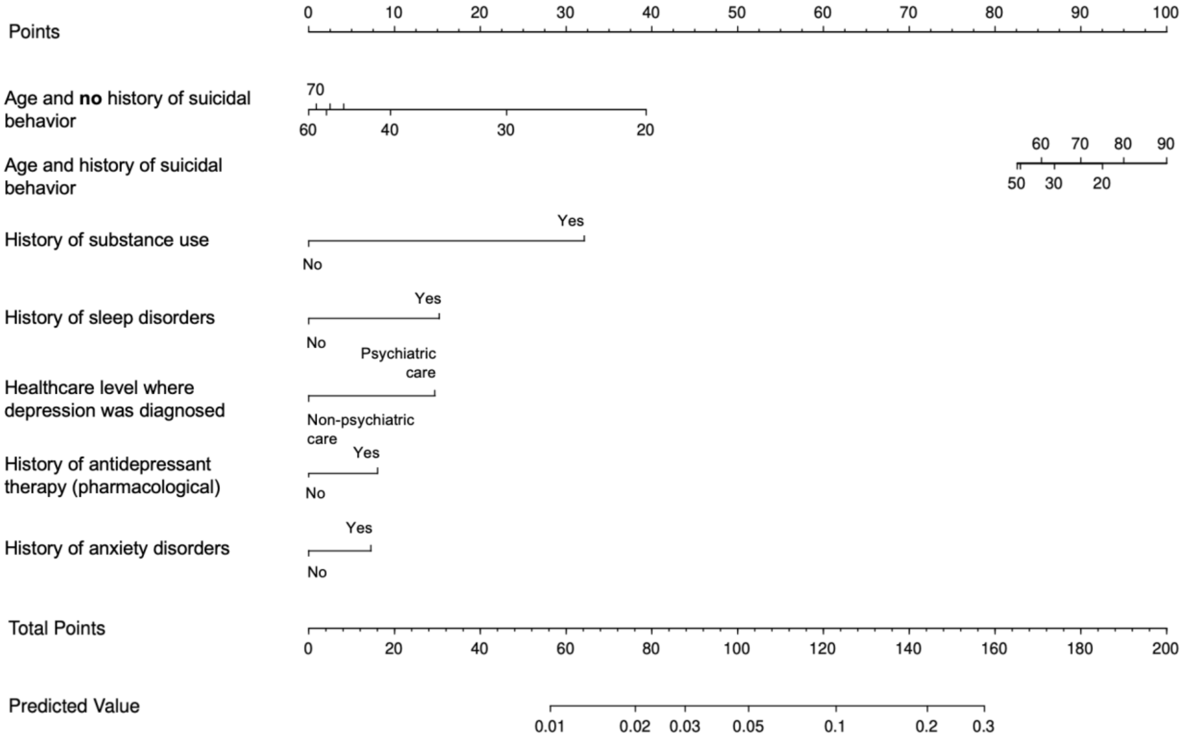

[a] A Cox's proportional hazard model including all variables (see Supplement D) was fitted to the data. Possible non-linearities were handled by transforming the continuous variables using restricted cubic splines, each with three knots placed at the respective 10th, 50th, and 90th sample percentiles. We approximated the full model by using a fast backward algorithm on an ordinary least squares model in which the estimated linear predictor from the full model was the outcome and all variables were entered in exactly the same manner as in the full model. Thus, in the first step $R^2 = 1.0$ by design and by removing variables in a stepwise manner, the full model could be approximated to an arbitrary level. The model was internally validated using 300 bootstrap samples. In validation of the final model, we accounted for non-independence (i.e., that one individual could have had more than one SB/MDD-episode). Discrimination was assessed by Harrell's *c*-index and calibration was assessed graphically by comparing observed event rates with the predicted risk at one year after index.

[b] Instructions on how to interpret the nomogram using an example: The risk for SB within one year after start of MDD-episode for a 40-year-old patient with no history of SB, but with a history of substance use and sleep disorders, who was diagnosed in non-psychiatric care, with a history of AD-therapy but no history of anxiety, is arrived at as follows. Start with the line "age and no history of SB" and mark "40". This mark is compared to the first row, by drawing a straight vertical line, to get the risk point of approximately 10. The row "history of substance use" is "yes" for the patient, which corresponds to around 32 risk points (first row) and "history of sleep disorders" is "yes" which corresponds to about 15 risk points. "History of antidepressant therapy" was "yes" for the patient, which corresponds to approximately 8 risk points, and the rows "healthcare level where depression was diagnosed" and "history of anxiety disorders" both correspond to 0 risk points (as the patient had "no" on these two variables). Finally, all risk points are summed-up (i.e., 10+32+15+0+8+0 in this example) rendering in a total risk score (the row "total points") of 65. This sum is compared to the lowest row "predicted value", which is equivalent to a risk for SB just above 0.015 (1.5%).

[c] Suicidal behavior (SB) was defined by the ICD10 codes X60-X84 (intentional self-harm) recorded in any diagnosis position and in both outpatient and inpatient healthcare settings. See methods section for more information.

[d] Definition of a major depressive disorder (MDD) episode is displayed in Supplement A.

[e] All covariates used in the development of the model are based on data during a time period of three years before start of the MDD-episode and defined in Supplement D.

© 2023 Lundberg J et al. *JAMA Psychiatry.*

eFigure 7. Calibration of the Cox Proportional Hazards Model on Determinants for Suicidal Behavior (SB)[a] Within 1 Year After Start of a Major Depressive Disorder (MDD) Episode[b]

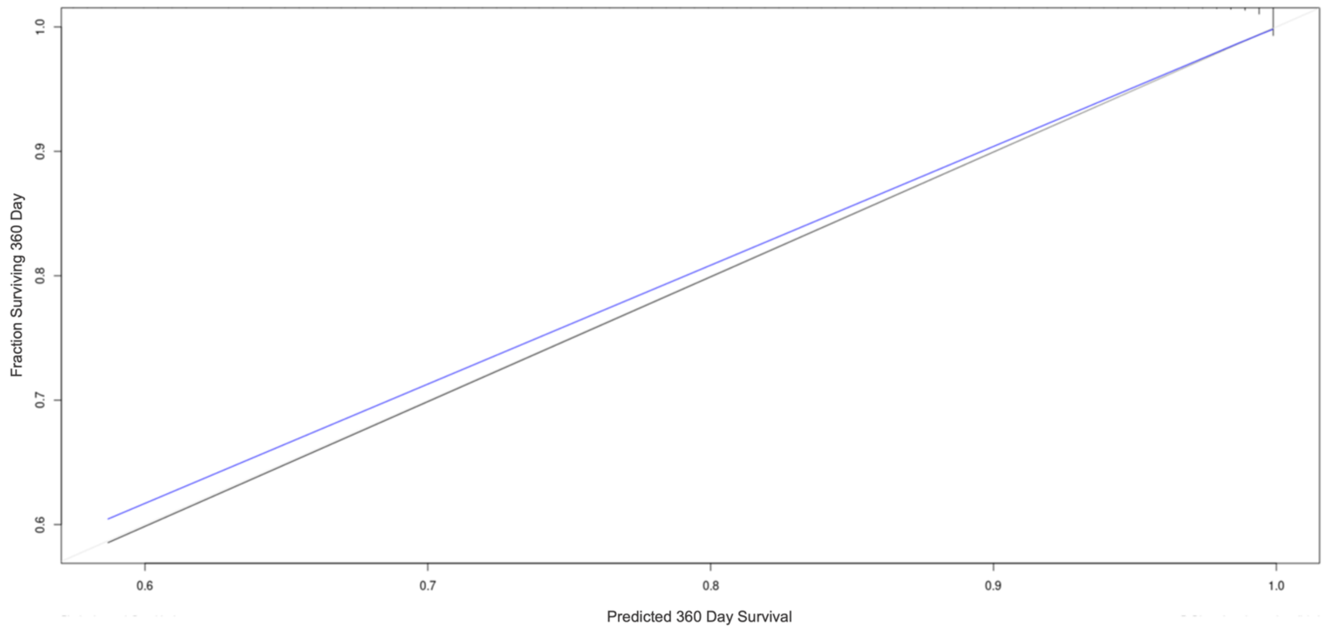

[a] Suicidal behavior (SB) was defined by the ICD10 codes X60-X84 (intentional self-harm) recorded in any diagnosis position and in both outpatient and inpatient healthcare settings. See methods section for more information.

[b] Definition of a major depressive disorder (MDD) episode is displayed in Supplement A.

© 2023 Lundberg J et al. *JAMA Psychiatry.*
