# Supplementary material for: Determinants and Outcomes of Suicidal Behavior Among Patients With Major Depressive Disorder
Source: JAMA Psychiatry. 2023 Aug 16;80(12):1218–25. doi: 10.1001/jamapsychiatry.2023.2833 (PMC10433143; doi:10.1001/jamapsychiatry.2023.2833)
Supplement: Supplement 2. — Data Sharing Statement [file jamapsychiatry-e232833-s002.pdf]

Data Sharing Statement

Lundberg. Determinants and Outcomes of Suicidal Behavior Among Patients With Major Depressive Disorder. *JAMA Psychiatry*. Published August 16, 2023.
doi:10.1001/jamapsychiatry.2023.2833

Data
Data available: No

Additional Information
Explanation for why data not available: Data for this research project will be available after approval of proposal, and after a legal assessment with regards to confidentiality of the data. Requests, including study rationale, should be sent to corresponding author.
